# Supplementary material for: The Characteristics of Tumor Microenvironment Predict Survival and Response to Immunotherapy in Adrenocortical Carcinomas
Source: Cells. 2023 Feb 27;12(5):755. doi: 10.3390/cells12050755 (PMC10000893; doi:10.3390/cells12050755)
Supplement: Supplementary file 1 [file cells-12-00755-s001.zip › Supplementary figures.pdf]

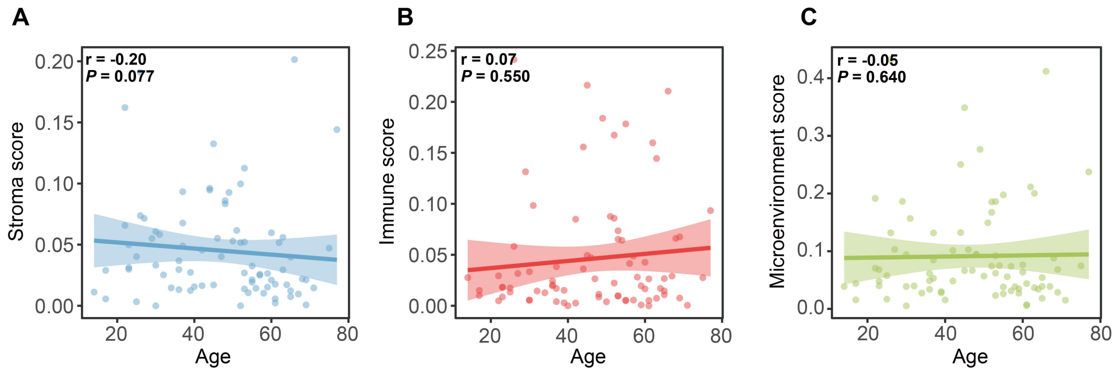

Figure S1. Spearman analysis of the correlation between TME-related scores and age. (A) Correlation of stromal score with age. (B) Correlation of immune score with age. (C) Correlation of microenvironmental scores with age.

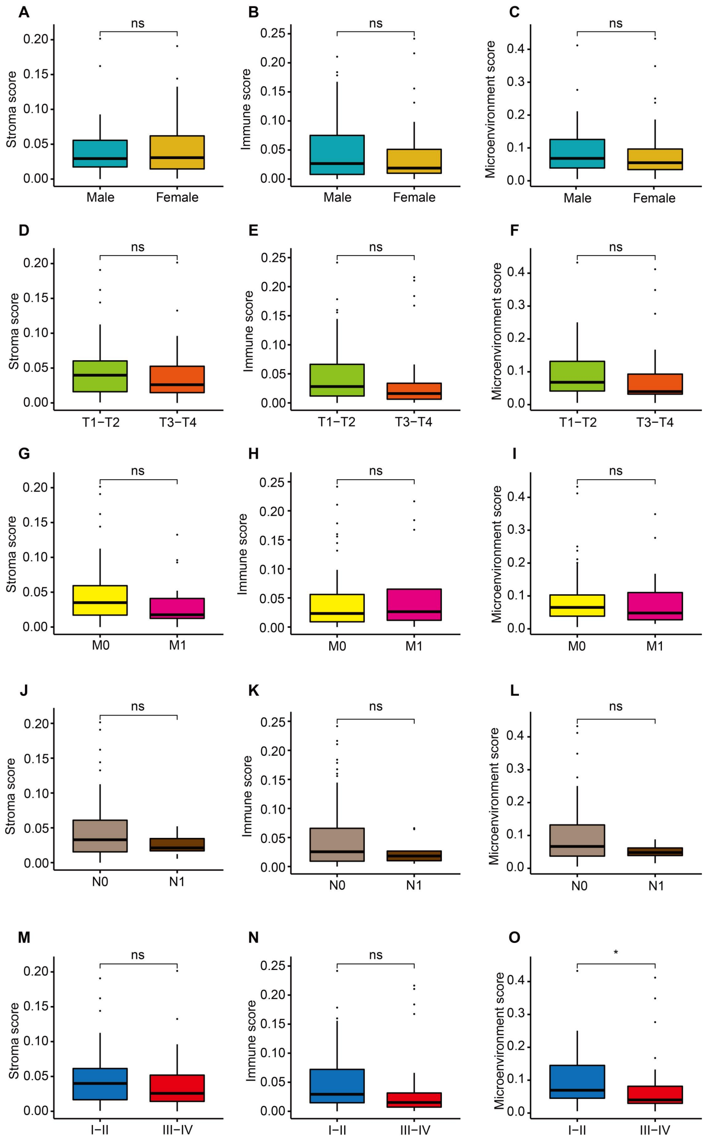

Figure S2. TME-related scores and five clinical classification characteristics. (A-C) Comparison of differences in TME-related scores between different gender groups. (D-F) Comparison of the differences in TME-related scores between different T stage groups. (G-I)

Comparison of differences in TME-related scores between different M stage groups. (J-L) Comparison of differences in TME-related scores between different N stage groups. (M-O) Comparison of differences in TME-related scores between different tumor stage groups. Data were analyzed by Wilcoxon test; ns no significance, *$p < 0.05$.

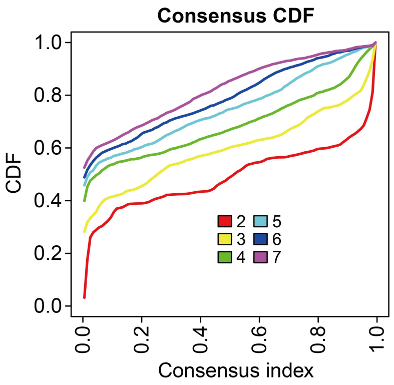

Figure S3. Change in consistency index versus CDF size when K = 2-7.

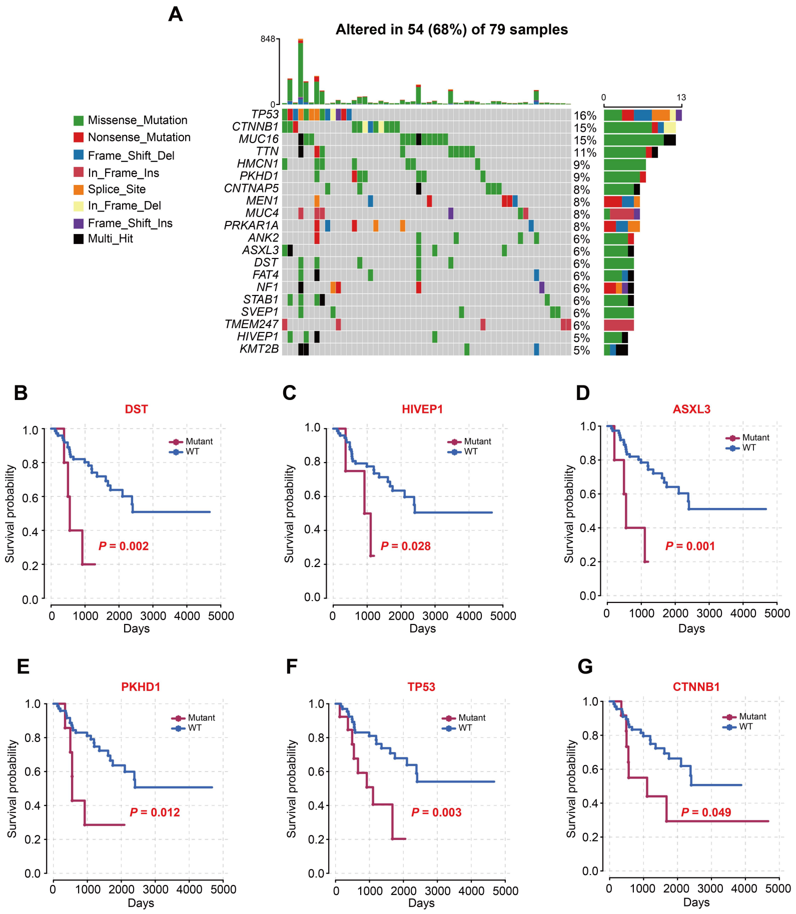

Figure S4. Somatic mutational analysis in ACC. (A) Waterfall plot to visualize the distribution of the top 20 highly mutated genes. (B-G) Association of mutations in genes DST, HIVEP1, ASXL3, PKHD1, TP53 and CTNNB1 with OS.

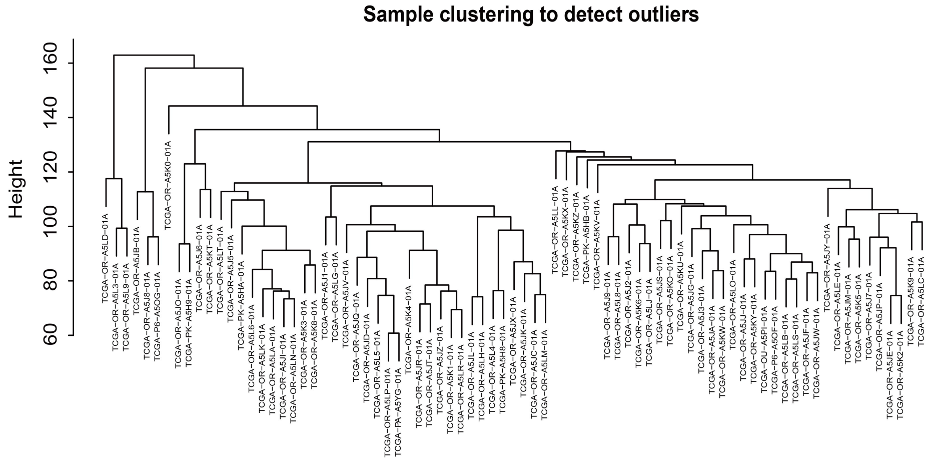

Figure S5. Sample hierarchical clustering trees were drawn based on 5000 high MAD genes and used to detect the presence of significant outliers.

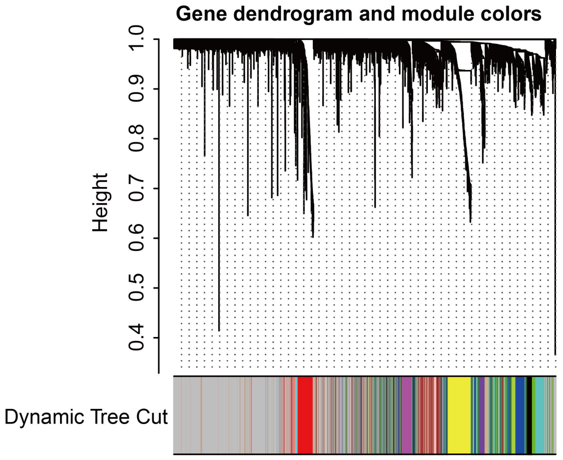

Figure S6. Hierarchical clustering of genes and gene modules under dynamic tree cut method.

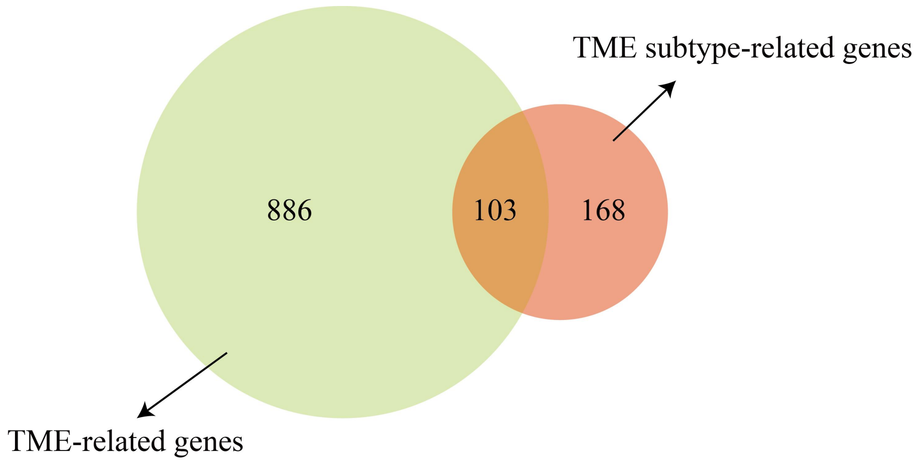

Figure S7. Venn diagram visualization of the intersection of TME-related genes with TME-related subtype genes.
